# Supplementary material for: Knowledge, attitudes, and practices (KAP) towards COVID-19 pandemic among pregnant women in a tertiary hospital in Karachi, Pakistan
Source: PLoS One. 2022 Nov 30;17(11):e0274252. doi: 10.1371/journal.pone.0274252 (PMC9710773; doi:10.1371/journal.pone.0274252)
Supplement: Attachment — Submitted filename: RESPONSE TO REVIEWER.docx [file pone.0274252.s002.docx]

**RESPONSE TO REVIEWER**

1. Please ensure that your manuscript meets PLOS ONE's style requirements, including those for file naming. The PLOS ONE style templates can be found at

Manuscript style including file name is corrected

2. Please include additional information regarding the survey or questionnaire used in the study and ensure that you have provided sufficient details that others could replicate the analyses. For instance, if you developed a questionnaire as part of this study and it is not under a copyright more restrictive than CC-BY, please include a copy, in both the original language and English, as Supporting Information. If the original language is written in non-Latin characters, for example Amharic, Chinese, or Korean, please use a file format that ensures these characters are visible.

Data was collected on the self-administered questionnaire, developed after a literature review and from WHO recommendation [^[1]^]. The content of the questionnaire was grouped into various themes, including demographic characteristics, obstetric variables,	knowledge, attitude, and practice of COVID-19-related questions.

3. Please state whether you validated the questionnaire prior to testing on study participants. Please provide details regarding the validation group within the methods section.

A pretest was conducted to ascertain the validity of the questionnaire. Mentioned in manuscript

4. In your Data Availability statement, you have not specified where the minimal data set underlying the results described in your manuscript can be found. PLOS defines a study's minimal data set as the underlying data used to reach the conclusions drawn in the manuscript and any additional data required to replicate the reported study findings in their entirety. All PLOS journals require that the minimal data set be made fully available. For more information about our data policy, please see [http://journals.plos.org/plosone/s/data-availability](https://eur03.safelinks.protection.outlook.com/?url=http%3A%2F%2Fjournals.plos.org%2Fplosone%2Fs%2Fdata-availability&data=04%7C01%7Cdur.shahwar%40aku.edu%7Cd2dedea97a3645c98ef808d9c5d8c265%7Ca5d4252a02f94e6096f09733baae4919%7C0%7C0%7C637758357939000816%7CUnknown%7CTWFpbGZsb3d8eyJWIjoiMC4wLjAwMDAiLCJQIjoiV2luMzIiLCJBTiI6Ik1haWwiLCJXVCI6Mn0%3D%7C3000&sdata=zJrK2PtavrTnpLq955uOFe22nATUR3Ism1Jt4pay%2FRg%3D&reserved=0).

Link of Data set is created in Manuscript before references

"Upon re-submitting your revised manuscript, please upload your study’s minimal underlying data set as either Supporting Information files or to a stable, public repository and include the relevant URLs, DOIs, or accession numbers within your revised cover letter. For a list of acceptable repositories, please see [http://journals.plos.org/plosone/s/data-availability#loc-recommended-repositories](https://eur03.safelinks.protection.outlook.com/?url=http%3A%2F%2Fjournals.plos.org%2Fplosone%2Fs%2Fdata-availability%23loc-recommended-repositories&data=04%7C01%7Cdur.shahwar%40aku.edu%7Cd2dedea97a3645c98ef808d9c5d8c265%7Ca5d4252a02f94e6096f09733baae4919%7C0%7C0%7C637758357939000816%7CUnknown%7CTWFpbGZsb3d8eyJWIjoiMC4wLjAwMDAiLCJQIjoiV2luMzIiLCJBTiI6Ik1haWwiLCJXVCI6Mn0%3D%7C3000&sdata=TPD%2BUD636eio%2FwrEqNtYWn8JuB1kZ5YXHRHN5UELpIk%3D&reserved=0). Any potentially identifying patient information must be fully anonymized.

Important: If there are ethical or legal restrictions to sharing your data publicly, please explain these restrictions in detail. Please see our guidelines for more information on what we consider unacceptable restrictions to publicly sharing data: [http://journals.plos.org/plosone/s/data-availability#loc-unacceptable-data-access-restrictions](https://eur03.safelinks.protection.outlook.com/?url=http%3A%2F%2Fjournals.plos.org%2Fplosone%2Fs%2Fdata-availability%23loc-unacceptable-data-access-restrictions&data=04%7C01%7Cdur.shahwar%40aku.edu%7Cd2dedea97a3645c98ef808d9c5d8c265%7Ca5d4252a02f94e6096f09733baae4919%7C0%7C0%7C637758357939010807%7CUnknown%7CTWFpbGZsb3d8eyJWIjoiMC4wLjAwMDAiLCJQIjoiV2luMzIiLCJBTiI6Ik1haWwiLCJXVCI6Mn0%3D%7C3000&sdata=iqrncOo5snUlbPnPP0u4HZm4JmN3ABwbG1ynvEtcxI8%3D&reserved=0). Note that it is not acceptable for the authors to be the sole named individuals responsible for ensuring data access.

We will update your Data Availability statement to reflect the information you provide in your cover letter.

5. Please include your full ethics statement in the ‘Methods section of your manuscript file. In your statement, please include the full name of the IRB or ethics committee who approved or waived your study, as well as whether or not you obtained informed written or verbal consent. If consent was waived for your study, please include this information in your statement as well.

Data was collected after approval of the ethical review committee (Reference number; 2020-4976-14204) of (Aga khan university hospital (AKUH) Karachi, Pakistan. Anonymity and confidentiality of data were maintained.

Data were collected during the full quarantine in March and April 2020.

Pregnant patient attending obstetric clinics for consultation were asked to participation in the study, who agreed for participation an informed consent in writing has been taken before completing questionnaire.

6. Please include a separate caption for each figure in your manuscript.

Incorporated in manuscript

7. Please ensure that you refer to Figure 2 in your text as, if accepted, production will need this reference to link the reader to the figure.

Incorporated in manuscript

8. Please include your tables as part of your main manuscript and remove the individual files. Please note that supplementary tables (should remain/ be uploaded) as separate "supporting information" files".

9. We note you have included a table to which you do not refer in the text of your manuscript. Please ensure that you refer to Table 1 in your text; if accepted, production will need this reference to link the reader to the Table.

Incorporated in manuscript

[Note: HTML markup is below. Please do not edit.]

Reviewers'comments:

Reviewer's Responses to Questions

**Comments to the Author**

1. Is the manuscript technically sound, and do the data support the conclusions?

The manuscript must describe a technically sound piece of scientific research with data that supports the conclusions. Experiments must have been conducted rigorously, with appropriate controls, replication, and sample sizes. The conclusions must be drawn appropriately based on the data presented.

Reviewer #1: Partly

Reviewer #2: Yes

2. Has the statistical analysis been performed appropriately and rigorously?

Reviewer #1: No

Reviewer #2: Yes

5. Have the authors made all data underlying the findings in their manuscript fully available?

Provided in Covering letter and as a supplementary file with name of S1 Data sheet

The [PLOS Data policy](https://eur03.safelinks.protection.outlook.com/?url=http%3A%2F%2Fwww.plosone.org%2Fstatic%2Fpolicies.action%23sharing&data=04%7C01%7Cdur.shahwar%40aku.edu%7Cd2dedea97a3645c98ef808d9c5d8c265%7Ca5d4252a02f94e6096f09733baae4919%7C0%7C0%7C637758357939010807%7CUnknown%7CTWFpbGZsb3d8eyJWIjoiMC4wLjAwMDAiLCJQIjoiV2luMzIiLCJBTiI6Ik1haWwiLCJXVCI6Mn0%3D%7C3000&sdata=mpNEVaLWXBQsmWIurTLiPwWX9Hn8ckY%2FQdu%2FGs%2BPPqg%3D&reserved=0) requires authors to make all data underlying the findings described in their manuscript fully available without restriction, with rare exception (please refer to the Data Availability Statement in the manuscript PDF file). The data should be provided as part of the manuscript or its supporting information, or deposited to a public repository. For example, in addition to summary statistics, the data points behind means, medians and variance measures should be available. If there are restrictions on publicly sharing data—e.g. participant privacy or use of data from a third party—those must be specified.

Reviewer #1: No

Reviewer #2: Yes

4. Is the manuscript presented in an intelligible fashion and written in standard English?

PLOS ONE does not copyedit accepted manuscripts, so the language in submitted articles must be clear, correct, and unambiguous. Any typographical or grammatical errors should be corrected at revision, so please note any specific errors here.

Reviewer #1: No

Reviewer #2: Yes

5. Review Comments to the Author

Please use the space provided to explain your answers to the questions above. You may also include additional comments for the author, including concerns about dual publication, research ethics, or publication ethics. (Please upload your review as an attachment if it exceeds 20,000 characters)

Reviewer #1: 1. Sample size calculated by WHO calculator is 380 assuming a response rate of 50%, confidence interval (CI) 95%, Z as 1.96, and margin of error d as 5% by assuming 5000 deliveries occurred per annum in obstetrics and gynecology unit as per unit annual statistics – VERIFY AND INCLUDE THE CALCULATION IN THE TEXT

1. The sample size calculated by the WHO calculator is 360 assuming a response rate of 50%, confidence interval (CI) of 95%, Z as 1.96, and margin of error d as 5% by assuming 5000 deliveries occurred per annum in obstetrics and gynecology unit as per unit annual statistics. Hence, the sample size was n= (Z) ^2^ P (1-P) N /d^2^ (N-1) + (Z) ^2^ P (1-P), by considering the incomplete responses, we included the target sample of 380. 2.
2. Explain the sampling method

Non-Probability convenience sampling method

3. Individual scoring of 10 and above were categorized as excellent whereas score below 10 were considered as poor knowledge – HOW WAS THE CUT OFF DETERMINED

Above and equal to 80% (10/12) was considered excellent to good knowledge**.**

4. QUESTIONNAIRE – 21 ITEM QUESTIONNAIRE – Explain the validation process

The validity of content was reviewed for appropriateness, relevance, applicability, and accuracy by two senior faculty members, epidemiologist and educationist. Long statements were rephrased and make them clear, and unambiguous. Items that need exclusion were highlighted and removed.

5. The attitude section consisted of three questions, and the response of each item collected on a 3 point Likert scale as follows 0 (“Disagree”), 1 (“Undecided”), and 2 (“Agree”) – UNDECIDED CATEROGRY IN LIKERT SCALE IS NOT UNIVERSALLY FOLLOWED, BETTER TO BE CONVERTED AS NEAUTRAL

UNDECIDED have been replaced into Neutral

6. The total score ranged from 0 to 6 with an overall greater score indicates more positive attitudes towards the COVID-19. A cut off level of 6 was set for more positive attitudes towards the prevention of COVID infection – HOW WAS CUT OFF ARRIVED?

Above and equal to 80% (5/6) was considered good attitude.

7. The reliability of the questionnaire checked by conducting a pretest among pregnant women by taking 5% of the sample size. From the pretest, understandability, clarity, and organization of the questionnaire will be checked and reviewed – IF RELIABALITY WAS DONE – WHATS THE CRONBACH’S ALPHA VALUE AND ITEM ANALYSIS TO BE EXPLAINED

**Incorporated in methodology**

Cronbach’s Alpha for Knowledge = 0.93 (12 item)

Cronbach’s Alpha for attitude = 0.98 (3 item)

Cronbach’s Alpha for practice = 0.85(6 item)

8. STUDY TOOL – The 21 item questionnaire – Validation process is not explained in the article.

**Same as question 4**

9. Data was entered and analyzed using SPSS version 21. Data was analyzed by using SPSS 19. – DESCRIBE THE VERSION CLEARLY AND MENTION THE LICENSE AGREEMENT

SPSS 19 version Used

10. Calculated sample size is 380, but the results were given only for 377 due to 3 missing data which is less than optimum sample size – Author has to view this seriously as sampling and sample size determines the internal and external validity of any quantitative study.

**Also Incorporated in manuscripts**

The sample size calculated by the WHO calculator is 360 assuming a response rate of 50%, confidence interval (CI) of 95%, Z as 1.96, and margin of error d as 5% by assuming 5000 deliveries occurred per annum in obstetrics and gynecology unit as per unit annual statistics. Hence, the sample size was n= (Z) ^2^ P (1-P) N /d^2^ (N-1) + (Z) ^2^ P (1-P), by considering the incomplete responses, we included the target sample of 380.

Reviewer#2: Summary:
The study is a well-grounded scientific research to assess the Knowledge, attitude and practice regarding COVID 19 among pregnant mothers. The findings of the study will be useful in identifying and filling the gaps in knowledge regarding COVID 19 among pregnant mothers.

Background:
1. WHO, ARDS and KAP has to be expanded for the first time in the manuscript before using them as abbreviations thereafter.

Corrected

2. Since study has been completed the phrase “study has been planned” can be avoided. Instead “Study was done” can be used.

Corrected

3. Grammatical errors are present throughout the background section which needs to be addressed.

Corrected
4. COVID – 19 has to be represented uniformly in capital letters throughout the manuscript.

Corrected

5. The Justification for the study could be explained in more detail with detailed statistics regarding COVID 19 related deaths among preganant mothers - Globally, Nationally and in the area of study.

Mentioned in manuscript

Methods:
1. More explanation is required regarding the sample size calculation. (Eg: Reference for WHO sample size calculator)

Mentioned above and Incorporated in manuscript.

2. Since the questionnaire is self-made and not standardized, Cronbach alpha and rationale behind choosing the cut-off has to be mentioned in the manuscript. The author could explain whether they used median/mean or interquartile range of the scores to choose the cut-offs.

Incorporated in manuscript

3. In page 5, line 115, terms like “will be checked and reviewed” has to be avoided as the study is completed.

Done

4. Sampling method and study duration are not mentioned in the methods section.

Mentioned above and in manuscript

5. The author could provide details regarding the language in which questionnaire was used.

The questionnaire was prepared in English and then translated into the Urdu language with the assistance of language experts. Mentioned in manuscript

6. Details regarding the mode of interview could be provided by the author. They could mention whether the participants filled on their own or an investigator asked questions and filled the form.

Participant filled themselves. And one of the team members was available to facilitate the patients in case of queries related to the questionnaire. Mentioned in manuscript

Results:
1. In the results section, Table 1 could to be cited in Page 6, Line no:115 to give readers that the details are present in frequency table.

Corrected in manuscript
2. In figure 1, the numbers looks pixelated and not visible. The authors could provide an image with good clarity so that, the numbers looks readable.

Corrected in manuscript

3. The authors could give details regarding the participants who gave no answer in attitude and practice section and how those responses were used in analysis.

Participant who did not respond were used in denominator in analysis and listed in table 3. For attitude, we observed 9 participants in which 4 did not respond in all three questions and five participants did not response 1 out of 3 questions of attitude.

For Practice, we observed three participants in which two participants did not response 1 out of 5 questions and one participants did not response 2 out of 5 question. See table 3, we included in the analysis

4. The authors could interpret the beta coefficients in details to add value to the interpretation of the findings.

**Following lines also incorporated in results text**

In Univariate analysis, regression coefficient showed that woman with above 30 years of age(vs. ≤ 30, β= -2.87, p<0.01), grand multiparty (vs. no parity, β= -2.50, p<0.01), working women (vs. housewife, β= -4.29, p<0.01) and those women who had got information by internet ( vs. others, β = -3.75, p<0.01) were significantly associated with low knowledge. In multivariate analysis, adjusted regression coefficient (by general linear model) showed that above 30 years (vs. ≤ 30, β= -0.96, p<0.01) , less family member<4 (vs. >7, β= -0.89, p<0.001), working women (vs. housewife, β= -1.58, p<0.01) and those women who had got information by internet ( vs. others, β = -2.39, p<0.01) were significantly associated with lower knowledge score (table 4). Corrected in manuscript also.

5. In table 5, since 0 is present in many of the cells, the authors could give details on the statistical test in the univariate analysis used to establish association between variables

Corrected in manuscript: Chi-square test or fisher’s exact test was used for Univariate analysis. . P≤0.05 was considered as significant**.**

Discussion:
1. Repetition of findings mentioned in the results could be avoided in the discussion. Instead a gist can be given and discussed in detail with relevant study comparisons.

Corrected in manuscript

6. PLOS authors have the option to publish the peer review history of their article ([what does this mean?](https://eur03.safelinks.protection.outlook.com/?url=https%3A%2F%2Fjournals.plos.org%2Fplosone%2Fs%2Feditorial-and-peer-review-process%23loc-peer-review-history&data=04%7C01%7Cdur.shahwar%40aku.edu%7Cd2dedea97a3645c98ef808d9c5d8c265%7Ca5d4252a02f94e6096f09733baae4919%7C0%7C0%7C637758357939020800%7CUnknown%7CTWFpbGZsb3d8eyJWIjoiMC4wLjAwMDAiLCJQIjoiV2luMzIiLCJBTiI6Ik1haWwiLCJXVCI6Mn0%3D%7C3000&sdata=nIbfwKP01zvxqdZsmNTXqos5YeA6aH4c42sxENE7zvY%3D&reserved=0)). If published, this will include your full peer review and any attached files.


If you choose “no”, your identity will remain anonymous but your review may still be made public.


**Do you want your identity to be public for this peer review?** For information about this choice, including consent withdrawal, please see our [Privacy Policy](https://eur03.safelinks.protection.outlook.com/?url=https%3A%2F%2Fwww.plos.org%2Fprivacy-policy&data=04%7C01%7Cdur.shahwar%40aku.edu%7Cd2dedea97a3645c98ef808d9c5d8c265%7Ca5d4252a02f94e6096f09733baae4919%7C0%7C0%7C637758357939020800%7CUnknown%7CTWFpbGZsb3d8eyJWIjoiMC4wLjAwMDAiLCJQIjoiV2luMzIiLCJBTiI6Ik1haWwiLCJXVCI6Mn0%3D%7C3000&sdata=PGfJxSLVRmOxTiqoo2gXFAGYTkXbBsK%2FwWOKnTI%2FH1c%3D&reserved=0).

Reviewer #1: No

Reviewer #2: No

[NOTE: If reviewer comments were submitted as an attachment file, they will be attached to this email and accessible via the submission site. Please log into your account, locate the manuscript record, and check for the action link "View Attachments". If this link does not appear, there are no attachment files.]

While revising your submission, please upload your figure files to the Preflight Analysis and Conversion Engine (PACE) digital diagnostic tool, [https://pacev2.apexcovantage.com/](https://eur03.safelinks.protection.outlook.com/?url=https%3A%2F%2Fpacev2.apexcovantage.com%2F&data=04%7C01%7Cdur.shahwar%40aku.edu%7Cd2dedea97a3645c98ef808d9c5d8c265%7Ca5d4252a02f94e6096f09733baae4919%7C0%7C0%7C637758357939030796%7CUnknown%7CTWFpbGZsb3d8eyJWIjoiMC4wLjAwMDAiLCJQIjoiV2luMzIiLCJBTiI6Ik1haWwiLCJXVCI6Mn0%3D%7C3000&sdata=mMKE0fXiHWYwjlbpMv671lMHvnNjrbPL2L%2BIyGhkRXc%3D&reserved=0). PACE helps ensure that figures meet PLOS requirements. To use PACE, you must first register as a user. Registration is free. Then, login and navigate to the UPLOAD tab, where you will find detailed instructions on how to use the tool. If you encounter any issues or have any questions when using PACE, please email PLOS at [figures@plos.org](mailto:figures@plos.org). Please note that Supporting Information files do not need this step.

Corrected in manuscript.
